# *α*-Tocopherol Protects the Heart, Muscles, and Testes from Lipid Peroxidation in Growing Male Rats Subjected to Physical Efforts

**DOI:** 10.1155/2019/8431057

**Published:** 2019-07-24

**Authors:** Magdalena Górnicka, Anna Ciecierska, Jadwiga Hamulka, Małgorzata E. Drywień, Joanna Frackiewicz, Krzysztof Górnicki, Agata Wawrzyniak

**Affiliations:** ^1^Department of Human Nutrition, Faculty of Human Nutrition and Consumer Sciences, Warsaw University of Life Sciences, Warsaw 02-776, Poland; ^2^Department of Fundamental Engineering, Faculty of Production Engineering, Warsaw University of Life Sciences, Warsaw 02-787, Poland

## Abstract

The effect of *α*-tocopherol supplementation on adaptation to training is still equivocal. The aim of the study was to determine the effect of training and *α*-tocopherol supplementation on *α*-tocopherol and thiobarbituric acid reactive substance (TBARS) concentration in the rat liver, heart, muscles, and testes. Male Wistar rats (*n* = 32) were divided into four groups (nonsupplemented, not trained—C; nonsupplemented, trained—CT; supplemented, not trained—E; supplemented and trained—ET). During the 14-day experimental period, 2 mg/d of vitamin E as *α*-tocopherol acetate was administered to the animals (groups E and ET). Rats in the training group (CT and ET) were subjected to 15 minutes of treadmill running each day. The *α*-tocopherol levels in rat tissues were assessed using high-performance liquid chromatography (HPLC). Lipid peroxides were determined by TBARS spectrophotometric method. *α*-Tocopherol had a significant impact on *α*-tocopherol concentration in all tissues. Training increased the *α*-tocopherol concentration in the heart and muscles but reduced it in the liver. Training also caused increased lipid peroxidation in the muscles, heart, and testes; but a higher *α*-tocopherol content in tissues reduced the TBARS level. The main finding of the study is that impaired *α*-tocopherol status and its adequate intake is needed to maintain optimal status to prevent damage to the skeletal and cardiac muscles as well as the testes in growing individuals.

## 1. Introduction

Physical activity, as indicated by numerous studies, is beneficial for the body through its preventive and therapeutic action [1, 2]. Systematic, moderate physical activity helps to maintain good health, increase quality of life, and reduce the risk of diseases associated with an unhealthy lifestyle [3]. However, during intense training, cellular respiration intensity increases, which leads to the formation of free radicals [4]. Increased oxygen use by cells results in increased production of reactive oxygen species (ROS). Increased ROS levels in cellular and extracellular spaces, exceeding the level of antioxidants, are defined as oxidative stress [5].

It was reported that oxidative stress is involved in many physiological conditions (e.g., aging and exercise) as well as diseases (including inflammation, cardiovascular and neurodegenerative diseases, and cancer) [5, 6]. Free radicals contribute to the damage of lipids, proteins, and DNA in cellular structures, thus altering cell functioning, which can consequently contribute to tissue damage [7–9]. ROS are involved in chemical reactions which play an important role in metabolism but are also seen as factors that cause cell aging of living organisms resulting from oxidative changes [10].

High-intensity physical activity induces many metabolic, hormonal, and immunological reactions. Nevertheless, all forms of physical exercise, both aerobic and nonaerobic, contribute to the formation of free radicals and thus oxidative stress. There have been many studies describing the impact of physical activity on the formation of oxidative stress in the muscles that are most involved during physical training [11]. However, effort-induced reactive oxygen forms are also created in other tissues, such as the liver, testes, heart, and lungs [12–15]. Due to differences in metabolic processes, individual organs respond to the emerging ROS in various ways. As a result of oxidative stress induced by physical activity, enzymatic and nonenzymatic (e.g., vitamin E) mechanisms are activated to defend cells from the harmful effects of free radicals and increase antioxidant mobilization from lipophilic tissues [16, 17]. Endogenous and exogenous antioxidants play a huge role in protecting against oxidative stress. They remove oxygen free radicals by changing them into inactive substances, thus minimizing the chain reaction leading to changes in the cell.

*α*-Tocopherol belongs to the nonenzymatic antioxidant defence system, whose main function is to interrupt the lipid peroxidation reaction and protect against oxidative stress [18, 19]. By interrupting lipid peroxidation reactions and removing free oxygen radicals, fat-soluble *α*-tocopherol plays an important role in stabilizing cell membranes [13, 20, 21]. It has been shown that *α*-tocopherol plays an important role in the course of many processes, including gene transcription regulation and enzyme and molecule activity involved in cell signalling pathway regulation [22–24]. Furthermore, *α*-tocopherol also participates in the regulation of cell proliferation and differentiation, cell adhesion, and immune response processes and also has anti-inflammatory and antioxidant effects. In connection with the above, it plays an important role in the prevention and treatment of many diseases, including neurologic abnormalities and myopathy [25] as well as cancers [26]. Many studies have also shown that the antioxidant properties of *α*-tocopherol that protect DNA from damage are associated with oxidative stress formation. *α*-Tocopherol can inhibit free radicals that are responsible for the formation of chemical changes in the DNA structure [27]. TBARS concentration assay is used as a marker of oxidative stress or lipid peroxidation [28, 29]. Generally, TBARS is an indirect marker of oxidative stress, but it is a direct marker of lipid damage caused by increased oxygen consumption during exercise, and *α*-tocopherol has a crucial role, as an antioxidant protecting the lipids from peroxidation. Lipid peroxidation generates a complex variety of products that are commonly used to determine cell damage. One by-product of this cascade is malondialdehyde, which is known to attack DNA causing mutative damage [30]. Moreover, findings from both animal and human studies support the hypothesis that both regular physical activity and vitamin E supplementation are useful for reducing oxidative stress [31]. On the other hand, the effect of vitamin E supplementation on skeletal muscle adaptation to training due to hormesis theory (ROS as intracellular signalling molecules cause beneficial adaptation process) is still equivocal [32].

Thus, the aim of the study was to determine the effect of training and *α*-tocopherol supplementation on *α*-tocopherol as well as thiobarbituric acid reactive substance (TBARS) concentration in selected rat tissues (liver, heart, muscles, and testes). We hypothesized that the beginning period of training in growing individuals contributes to a reduction of *α*-tocopherol concentration and may increase lipid peroxidation.

## 2. Materials and Methods

### 2.1. Animal and Diet

**Figure 1:**
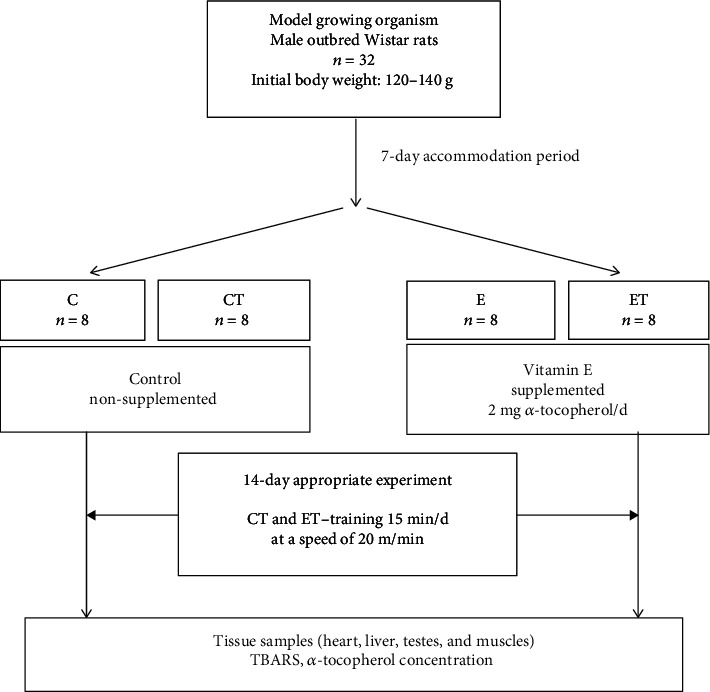
The study design. C: control group nonsupplemented with *α*-tocopherol, not trained; CT: control group nonsupplemented with *α*-tocopherol, trained; E: group supplemented with *α*-tocopherol, not trained; ET: group supplemented with *α*-tocopherol, trained.

Male outbred Wistar rats (*n* = 32) from certified breeding (Medical University of Białystok, Poland) with 120 to 140 g initial body weight were involved. After a 7-day adaptation, the animals were divided into 4 groups (*n* = 8) (Figure 1) and placed individually in plexiglass cages. More details about the conditions of keeping the animals was described in Wawrzyniak et al. [33]. The study obtained the approval of the III Local Animal Experiment Ethics Committee at the Warsaw University of Life Sciences (Resolution no. 33/2008).

During the appropriate experiment, lasting 14 days, and in the previous 7-day accommodation, the animals received diets in accordance with the AIN-93M diet [34] (components from Sigma-Aldrich, USA) (Table 1) deprived of *α*-tocopherol and had access to water.

Experimental diets were isocaloric and without vitamin E. Male Wistar rats (*n* = 32) were divided into four groups (nonsupplemented, not trained—C; nonsupplemented, trained—CT; supplemented, not trained—E; and supplemented and trained—ET). During the 14-day experimental period, 2 mg *α*-tocopherol acetate (in oil solution) was administered each day to the animals (groups E and ET), in drops per os, between 9:00 and 11:00 a.m., after exercise (groups ET and CT). Animals in the control groups (C, CT) received liquid drops deprived of *α*-tocopherol. Rats in the training groups (CT, ET) were subjected to 15 minutes of nonexhaustive treadmill running (20 m/min and 0° uphill slope) each day, to induce oxidative stress stimulated by physical effort.

### 2.2. Determination of Animal Development

To determine indicators of development of the experimental animals, the final and initial body weight was used (including the adaptation period), which allowed to calculate an increase in body weight gain during the experiment. Daily intake was also calculated. Moreover, the feed efficiency ratio (FER) was also calculated. FER is expressed by the ratio of body weight gain to 1 gram of the consumed diet [35]. The weights of the liver, heart, and testes were expressed using the somatic index, which determines the weight of the organ in relation to 100 grams of body weight [36].

### 2.3. Tissue Collection

At the end of the experiment, the rats were euthanized and tissues, such as the heart, liver, muscles, and testes, were taken. The tissues were rinsed in saline, dried on filter paper, weighed, frozen in liquid nitrogen, and stored at -80°C until analysis.

### 2.4. Analysis of *α*-Tocopherol in Tissues

The *α*-tocopherol content in rat tissues (liver, heart, muscles, and testes) was assessed using high-performance liquid chromatography with a UV-VIS detector. A LiChroCART®250-4 RP-18 (4 × 250 mm; 5 mm) with a precolumn (Merc, col. No. 841071, Darmstadt, Germany) was used. The weighed tissue samples were homogenized with hexane and ethanol. A 10% solution of vitamin C was also added to protect the *α*-tocopherol from oxidation. The prepared samples were then centrifuged at 2,500 rpm for 15 minutes at 4°C. After cooling, 100 *μ*L of the sample was taken and applied to the chromatography column on which the assay was made. The eluent was a mixture of acetonitrile, hexane, and isopropanol (65 : 14 : 21; *v*/*v*/*v*) and flow rate was 0.8 mL/min. The measurement was performed at a wavelength of UV 292 nm [37]. All the obtained results were applied to standard curves plotted for *α*-tocopherol (Sigma Company, St. Louis, MO, USA). The *α*-tocopherol concentration was expressed as nM/g of tissue.

### 2.5. Analysis of Lipid Peroxides

Lipid peroxide rat tissues (liver, heart, muscles, and testes) were determined by TBARS spectrophotometric method. This method is based on the measurement of the absorbance of the compound formed in the reaction of lipid oxidation products, mainly malondialdehyde (MDA) with thiobarbituric acid (TBA) [38], at a wavelength of 532 nm. The TBARS concentration was determined using a standard curve for which 1,1,3,3-tetraethoxypropane (TEP) was used. TBARS values were expressed as nM/g of tissue.

### 2.6. Statistical Analysis

Data were analysed using STATISTICA version 12. The results were presented as mean ± SD. Data distribution normality and homogeneity were checked using the Shapiro-Wilk test and were analysed by one-way ANOVA, two-way ANOVA, and a Tukey HSD post hoc test. The effects of *α*-tocopherol content on TBARS concentration in tissues were analysed with linear regression. For all analyses, statistical significance was considered at *α* = 0.05.

## 3. Results

### 3.1. The Impact of Training and *α*-Tocopherol Supplementation on Animal Development

The initial and final body weight as well as intake was not significantly different in all the experimental groups. However, statistically significant differences were observed in body weight gain as well as in the FER between the not trained control group (C) and the not trained, supplemented with *α*-tocopherol group (E) (Table 2). Body weight gain and FER were higher in group E compared to group C. This indicates that *α*-tocopherol caused faster weight gain in rats. Additionally, statistically higher FER values were also demonstrated in group E compared with the group supplemented with *α*-tocopherol and trained (ET) (Table 2). Significant impact of vitamin E supplementation on body weight gain and FER was noted (Table 2). However, no statistically significant effects were observed for training as well as the interaction between training and vitamin E supplementation on body weight gain and FER (Table 2).

There were statistically significant differences in liver and heart weight between group C and group E. Tissue weight was significantly higher in group E than in group C. In addition, there was also a higher heart weight in the ET compared with the control trained group (CT) (Table 3). However, there were no statistically significant differences in the somatic index for liver and testes in all experimental groups. Statistically significant differences in heart somatic index values between group C and group E as well as between CT and ET were found. The heart somatic index in group E was statistically higher than that in group C, and in the ET group than that in the CT group (Table 3). Vitamin E supplementation had a statistically significant effect on the liver and heart weight and also on the heart somatic index. However, neither training nor interaction of both training and vitamin E supplementation had a statistically significant effect on the weight of all examined organs and the somatic indexes (Table 3).

### 3.2. The Impact of Training and *α*-Tocopherol Supplementation on *α*-Tocopherol Content in Tissues

Table 4 shows *α*-tocopherol concentrations in selected rat tissues (liver, heart, muscles, and testes). The highest *α*-tocopherol concentrations (131.1 nM/g) were found in the liver, in the not trained supplemented group (group E); the lowest concentrations (9.39 nM/g) were in the muscles in the control group (group C).

There were statistically significant differences in *α*-tocopherol concentrations in the liver, heart, muscles, and testes between control groups (C vs. CT) and supplemented groups (E vs. ET) and between trained (CT vs. ET) and untrained groups (C vs. E). A statistically significantly higher *α*-tocopherol content was observed in all tissues in the CT group compared with the C group, as well as in the heart and muscles in the ET group compared with the E group. Moreover, a statistically significantly higher *α*-tocopherol content was found in all tissues in group E than in group C, and in group ET than in group CT (Table 4). Training had a significant effect on *α*-tocopherol concentration in the liver, heart, and muscles. *α*-Tocopherol supplementation had a significant effect on its concentration in all tissues. The results showed a significant impact both on training and *α*-tocopherol supplementation on *α*-tocopherol concentration in the testes (Table 4).

### 3.3. The Impact of Training and *α*-Tocopherol Supplementation on TBARS Concentrations in Tissues

Table 5 shows TBARS concentrations in selected rat tissues (liver, heart, muscles, and testes). There were statistically significant differences in TBARS concentrations in muscles and in testes between the control groups (C vs. CT). A statistically significantly higher TBARS concentration was observed in the heart in group CT compared with group ET (Table 5). Training had a significant effect on TBARS concentration in all tissues. *α*-Tocopherol supplementation had a significant effect on the TBARS concentration in the heart and muscles. The results showed a significant impact on both training and *α*-tocopherol supplementation on the TBARS concentration in the muscles and testes (Table 5).

### 3.4. Effects of *α*-Tocopherol Content on the TBARS Concentration in Tissues in Trained and Untrained Groups

**Figure 2:**
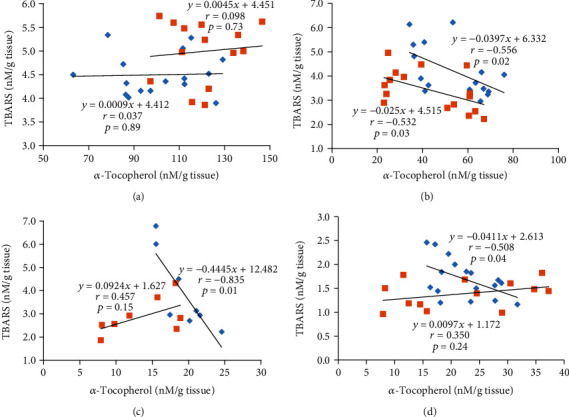
Effect of *α*-tocopherol content on TBARS concentration in the (a) liver, (b) heart, (c) muscles, and (d) testes; (blue diamond) trained groups (CT, ET); (red square) untrained groups (C, E).

No statistically significant correlation between *α*-tocopherol content and TBARS in the liver was observed (Figure 2). In the heart, a higher *α*-tocopherol content reduced the TBARS concentration in both trained and untrained groups (Figure 2). In trained groups, the *α*-tocopherol content in the muscles and testes was negatively associated with TBARS concentration (Figure 2).

## 4. Discussion

The study showed that (1) *α*-tocopherol supplementation significantly increases the liver and heart weight and heart somatic index; (2) *α*-tocopherol supplementation has a significant impact on its concentration in the liver, heart, muscles, and testes; (3) training increased the *α*-tocopherol concentration in the heart and muscles, but reduced it in the liver; and (4) training caused increased lipid peroxidation in the muscles, heart, and testes, but a higher *α*-tocopherol content in tissues reduced the TBARS level.

It is hard to compare our results with others due to the lack of similar studies.

The results indicate that *α*-tocopherol supplementation causes an increase in organ weight except for the liver. In turn, Kumar et al. [36] found that the heart somatic index in adult animals decreased significantly when supplemented with vitamin E. However, researchers speculate that a decreasing heart somatic index in animals receiving vitamin E may be due to an increase in body weight rather than a decrease in heart tissue. Vitamin E supplementation thus seems to be involved in increasing body weight with increased incorporation of vitamin E into the serum and heart tissues [36]. We suppose that *α*-tocopherol supplementation may contribute to increased body weight and organ weight in growing organisms. However, the association between vitamin E supplementation and body or organ weight requires further research.

As we noted, the highest mean *α*-tocopherol concentration was found in the liver, followed by the heart and the testes and was the lowest in the muscles. We found that *α*-tocopherol accumulates in all examined organs, which proves the effect of *α*-tocopherol supplementation on its increased content in the tissues. In the control groups (C, CT), *α*-tocopherol concentrations in all tissues were lower than in groups supplemented. Moreover, in the control group (C), which had a diet deprived of vitamin E, after 3 weeks we observed significantly decreased *α*-tocopherol concentrations in the liver. Vitamin E is mainly stored in adipose tissue and the liver, and supplementation primarily increases the *α*-tocopherol content in the liver, heart, spleen, and testes [39, 40]. Uchida et al. [41] observed a reduction in *α*-tocopherol concentrations in the liver after a week of a vitamin E-deficient diet, while its concentration in adipose tissue was unchanged even after 4 weeks, indicating that the liver is responsible for the rapid release of tocopherol reserves. The *α*-tocopherol content in tissues also reflects its ability to store *α*-tocopherol and the level of utilization of this compound. The low content of *α*-tocopherol in tissues probably results from the high *α*-tocopherol turnover or lower tissue capacity for its storage [42]. The liver participates in equalizing *α*-tocopherol concentrations when there is insufficient intake, which is probably explained by the highest *α*-tocopherol concentration in this tissue, determined on the basis of these results. The amount of tocopherol stored in the liver is the highest compared with other tissues [43, 44], which is consistent with our results. In our study, no significant correlation between *α*-tocopherol and TBARS concentration in the liver was observed. In opposite, Rodríguez-Gutiérrez et al. [29] and Ohta et al. [45] showed the ability of vitamin E to inhibit lipid peroxidation in the liver.

Our results indicated a significant effect of training on *α*-tocopherol concentration in the liver, heart, and muscles. We observed that training significantly reduced the *α*-tocopherol concentration in the liver and increased it in the heart and muscles in both the control and the supplemented groups. The study strengthens the evidence that during exercise, tocopherol is released from the liver, while in tissues such as the heart and muscles, in which the flow of blood and thus oxygen is increased, it is accumulated in larger quantities. The increase in *α*-tocopherol concentrations in the heart and muscles (induced by training) observed in our study can also be explained by the mobilization of liver reserves in the body. This may be confirmed by our early results that supplementation of *α*-tocopherol and training increased the plasma *α*-tocopherol concentration (results presented in Wawrzyniak et al. [46]). Intense exercise causes the mobilization of tocopherol from tissues into the bloodstream, which was confirmed by increased concentrations of *α*-tocopherol and triacylglycerols in plasma [47]. Training releases the *α*-tocopherol reserve in tissues and redistributes it between tissues [48] such as the heart and muscles, which are more involved during exercise. In studies with athletes supplemented with vitamin E, only the content in plasma of *α*-tocopherol was determined. The results showed that under the influence of physical effort the concentration of *α*-tocopherol did not change or increased due to vitamin E supplementation [49, 50]. We suppose that the determination of plasma *α*-tocopherol in training individuals may not be a good marker of nutritional status, because of its concentration increase in plasma, but with a decrease in other tissues. A possible explanation of these phenomena is that during intense skeletal muscle and myocardium exertion, *α*-tocopherol accumulated in other tissues is released due to the increased turnover of lipoproteins, causing the *α*-tocopherol content in the heart and muscles to be higher. Decreased *α*-tocopherol content in other organs, such as the liver and testes, may be caused by oxidative stress induced by physical effort. As an antioxidant, *α*-tocopherol is responsible for the removal of free radicals and increased lipid peroxidation can contribute to lowering its levels in tissues. The flow of blood in the heart and muscles increases during exercise and decreases in the liver and kidneys; hence, there may be different needs for antioxidants in the tissues [51]. It was indicated that supplementation with vitamin E was an important factor in the counteracting against oxidative stress and muscle damage [52, 53]. Our results clearly document that, compared with the liver or heart, skeletal muscles are more vulnerable to oxidative stress and need better antioxidant protection.

As we identified, training caused increased lipid peroxidation measures by the TBARS level in muscles and the heart. However, *α*-tocopherol supplementation reduced the TBARS level in muscle. Many studies have shown that training increases the TBARS level, which is a marker of lipid peroxidation in many tissues, such as the liver, skeletal muscle, kidneys, and heart [54]. A reduced muscle TBARS concentration and rate of muscle proteolysis as a result of vitamin E supplementation which prevented muscle atrophy was noted [55, 56]. In addition, an increased level of TBARS in muscles after acute exercise was demonstrated, although this increase was significantly suppressed by *α*-tocopherol supplementation in rats [57] and humans [58]. It was revealed that a deficiency of vitamin E in skeletal muscle is associated with an increase in contraction-mediated lipid peroxidation in skeletal muscle and a decrease in muscular force. Moreover, vitamin E deficiency impairs muscular endurance and alters muscle contractile properties following a prolonged series of contractions [59]. As a result of the imbalance between the production of ROS and the protective effect of antioxidants, the expression of transcription factors is modulated, leading to increased protein degradation, which results in muscle wasting [60]. Following the accumulation of ROS, mitogen-activated protein kinases (MAPKs), such as nuclear factor-*κ*B (NF-*κ*B) [61] as well as extracellular signal-regulated kinase (ERK) and p38, are activated [23, 62]. ERK has a direct effect on muscle cells and is responsible for the phosphorylation of specific transcription factors, which regulate the gene expression involved in basic cellular processes, such as proliferation, differentiation, and migration as well as cellular apoptosis. For muscles, it was shown that ROS induce the signalling cascade of the MAPK pathway, which resulted in changes in the mass and function of skeletal muscles and may contribute to muscular dystrophies and atrophies [62, 63]. NF-*κ*B participates in the regulation of multiple cellular processes related to proliferation, adhesion, migration, and viability. Moreover, it is also involved in inhibition of the skeletal myogenesis process and promoting proteolysis in skeletal muscle. Wang et al. [64] revealed that NF-*κ*B negatively regulates myofibrillar gene expression in proliferating myoblasts and also suppresses myogenesis by promoting the cell cycle and inhibiting MyoD synthesis. On the other hand, Mourkioti et al. [65], using an NF-*κ*B muscle-specific knockout mice model, revealed that inhibition of NF-*κ*B accelerated the process of muscle regeneration and enhanced muscle physical performance. Acute exercise activates NF-*κ*B [66] which, with MAPKs, may also be activated by tumor necrosis factor alpha (TNF-*α*) [67]. Moreover, Ladner et al. [68] showed that TNF-*α* inhibits myocyte differentiation by the activation of NF-*κ*B, in an in vitro study carried out on mouse C2C12 cells. However, it was shown that vitamin E supplementation improves muscle function and repairs skeletal muscle, reduces oxidative stress, and elevates antioxidant enzymes in vivo in rodents [69] and in vitro in C2C12 myoblasts [70]. Aoi et al. [57] found that *α*-tocopherol supplementation can attenuate inflammatory changes by reducing the activation of cytokines and adhesion molecules, and it also prevents muscle damage by attenuating the expression of NF-*κ*B. Huey et al. [71] also showed that vitamin E administration attenuates NF-*κ*B and TNF-*α* responses in skeletal and cardiac muscles which reduced muscle wasting.

We also found that higher *α*-tocopherol concentration was associated with lower lipid peroxidation level in the heart, regardless of training. Shekh and Mahmud [72] suggest that the combination of moderate exercise with *α*-tocopherol can be exploited to prevent atherosclerosis in hypercholesterolemic rabbits. However, Patil et al. [73] showed that antioxidant as *α*-tocopherol supplementation did not contribute significantly to improving the cardiopulmonary fitness of endurance athletes. These results indicate the participation of *α*-tocopherol in the protection of cellular components against the negative effects of increased free radical production and in counteracting oxidation processes intensified by intense physical exercise.

Our results provide strong evidence that training causes increased TBARS concentrations in the testes. Both *α*-tocopherol supplementation and training significantly affect the content of *α*-tocopherol in the testes, but a given *α*-tocopherol dose did not significantly reduce TBARS concentration. The protective effect of *α*-tocopherol supplementation on the reduction of morphological testicular alterations and favouring fertility has been demonstrated [74–76]. Burczynski et al. [77] described that the TBARS production rate by the testes, liver, and adrenal gland increased in *α*-tocopherol-deficient animals. Brezezińska-Slebodzińska et al. [78] described the reduced TBARS level in seminal plasma in boars due to vitamin E supplementation. Moreover, Surai et al. [40] in a study carried out on cockerels noted that the increased concentration of vitamin E in the testes and spermatozoa was associated with a reduction in their susceptibility to lipid peroxidation.

Adequate *α*-tocopherol intake is extremely important due to the decrease in the concentration of TBARS, and it is necessary to determine the dose which could unambiguously reduce the level of TBARS in all tissues and clearly contribute to reducing the oxidative stress level.

## 5. Conclusions

**Figure 3:**
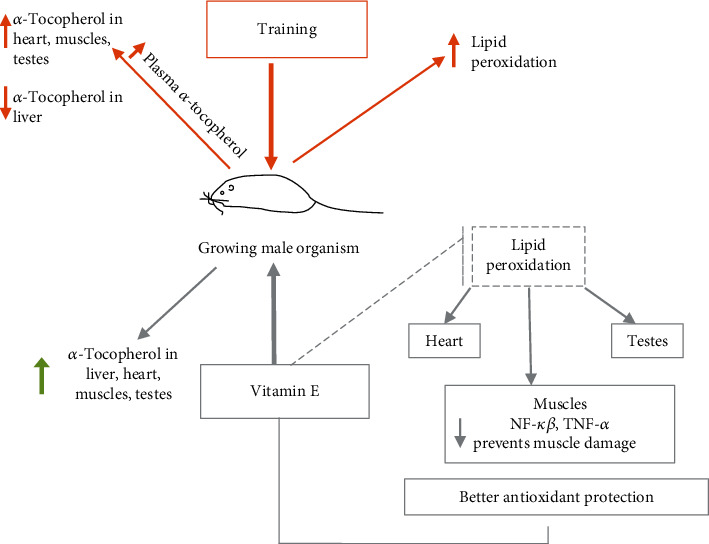
Conceptual illustration of the obtained results. NF-*κ*B: nuclear factor-*κ*B; TNF-*α*: tumor necrosis factor alpha.

The results obtained in our study indicate that *α*-tocopherol supplementation affected an increase in *α*-tocopherol content in rat tissues. Training caused an increase in *α*-tocopherol and TBARS concentration in the heart, muscles, and testes, while it reduced *α*-tocopherol in the liver. Comprehensive evaluation of *α*-tocopherol concentration in many tissues has clarified the mechanism of redistribution of *α*-tocopherol between tissues under the influence of training in growing males. The main finding of this study is that impaired *α*-tocopherol status and adequate intake of *α*-tocopherol is needed to maintain optimal status to prevent damage to the skeletal and cardiac muscles as well as the testes in growing individuals. Moreover, the results obtained in this study indicate that not higher than a physiological dose of *α*-tocopherol improved antioxidant protection in tissues by reducing the TBARS level (Figure 3). This suggests that adequate *α*-tocopherol intake is important for previously untrained young subjects and could provide health benefits and prevent against lipid peroxidation.

It needs to be highlighted that the determination of plasma *α*-tocopherol concentration in training individuals may be a weak biomarker of nutritional status or antioxidative defence, due to the plasma *α*-tocopherol increase as a result of training. This biomarker is often determined in research involving athletes, and its result may lead to an incorrect conclusion. In summary, our findings increased knowledge of the molecular mechanism involved in redistribution of *α*-tocopherol in training growing male organisms. Future research is needed to explain the observed effect of *α*-tocopherol supplementation on body gain. Our results may be also used to plan further research in the field of physical exercise with the simultaneous determination of more biomarkers associated with oxidative stress.

## Figures and Tables

**Table 1:** Composition of the experimental diet.

Ingredients	Quantity (g/kg)
Casein	140
Sucrose	100
Wheat starch	620
Cellulose	50
Soybean oil	40
Mineral mix^∗^	35
Vitamin mix without vitamin E^∗^	10
L-Methionine	1.8
Choline bitartrate	2.5
*t*-Butylhydroquinone	0.008

^∗^The mineral mix and the vitamin mix were prepared according to the AIN-93M diet [34].

**(a):** 

Development indicators	Groups				*p* value^1^ C vs. E	*p* value CT vs. ET
	Control		Vitamin E			
	C	CT	E	ET		
	*N* = 8	*N* = 8	*N* = 8	*N* = 8		
Body weight						
Initial (g)	152.2 ± 16.1	158.6 ± 15.4	154.2 ± 12.7	157.4 ± 17.3	NS	NS
*p* value	NS		NS			
Final (g)	251.3 ± 23.1	247.5 ± 21.2	264.1 ± 19.7	259.6 ± 15.5	NS	NS
*p* value	NS		NS			
Gain (g/d)	99.0 ± 12.7	89.0 ± 18.7	109.9 ± 8.81	102.1 ± 15.1	0.04	NS
*p* value	NS		NS			
Intake (g/d)	317.0 ± 39.5	299.7 ± 21.8	304.0 ± 19.3	317.3 ± 36.5	NS	NS
*p* value	NS		NS			
FER (g/g diet)	0.32 ± 0.05	0.30 ± 0.07	0.36 ± 0.02	0.32 ± 0.04	0.02	NS
*p* value	NS		0.02			

**(b):** 

	Two-way ANOVA, *p* value		
	Training	Vitamin E	Training × vitamin E
Body weight			
Initial (g)	NS	NS	NS
Final (g)	NS	NS	NS
Gain (g/d)	NS	0.01	NS
Intake (g/d)	NS	NS	NS
FER (g/g diet)	NS	0.04	NS

C: control group nonsupplemented with *α*-tocopherol, not trained; CT: control group nonsupplemented with *α*-tocopherol, trained; E: group supplemented with *α*-tocopherol, not trained; ET: group supplemented with *α*-tocopherol, trained; FER: feed efficiency ratio; ^1^Tukey HSD post hoc test, *p* value ≤0.05; NS: not significant.

**(a):** 

Development indicators	Groups				*p* value^1^ C vs. E	*p* value CT vs. ET
	Control		Vitamin E			
	C	CT	E	ET		
	*N* = 8	*N* = 8	*N* = 8	*N* = 8		
Liver						
Weight (g)	12.1 ± 1.85	12.3 ± 1.83	14.3 ± 2.61	13.2 ± 1.60	0.04	NS
*p* value	NS		NS			
Somatic index (%)	4.80 ± 0.51	4.96 ± 0.48	5.42 ± 0.87	5.08 ± 0.35	NS	NS
*p* value	NS		NS			
Heart						
Weight (g)	0.81 ± 0.05	0.79 ± 0.04	0.96 ± 0.10	0.96 ± 0.04	0.0005	<0.0001
*p* value	NS		NS			
Somatic index (%)	0.32 ± 0.03	0.32 ± 0.03	0.36 ± 0.04	0.37 ± 0.03	0.02	0.01
*p* value	NS		NS			
Testes						
Weight (g)	2.87 ± 0.27	2.97 ± 0.21	3.10 ± 0.63	3.36 ± 0.56	NS	NS
*p* value	NS		NS			
Somatic index (%)	1.15 ± 0.12	1.20 ± 0.07	1.17 ± 0.23	1.30 ± 0.21	NS	NS
*p* value	NS		NS			

**(b):** 

	Two-way ANOVA, *p* value		
	Training	Vitamin E	Training × vitamin E
Liver			
Weight (g)	NS	0.03	NS
Somatic index (%)	NS	NS	NS
Heart			
Weight (g)	NS	<0.0001	NS
Somatic index (%)	NS	0.0007	NS
Testes			
Weight (g)	NS	NS	NS
Somatic index (%)	NS	NS	NS

C: control group nonsupplemented with *α*-tocopherol, not trained; CT: control group nonsupplemented with *α*-tocopherol, trained; E: group supplemented with *α*-tocopherol, not trained; ET: group supplemented with *α*-tocopherol, trained; ^1^Tukey HSD post hoc test, *p* value ≤0.05; NS: not significant.

**(a):** 

*α*-Tocopherol (nM/g)	Groups				*p* value^1^ C vs. E	*p* value CT vs. ET
	Control		Vitamin E			
	C	CT	E	ET		
	*N* = 8	*N* = 8	*N* = 8	*N* = 8		
Liver	112.3 ± 11.4^a^	86.8 ± 11.6^b^	131.1 ± 10.7^b^	118.5 ± 7.40^a,b^	0.009	<0.0001
*p* value	0.0003		0.03			
Heart	27.2 ± 5.32^a^	40.4 ± 5.98^b^	59.6 ± 5.10^c^	67.1 ± 4.51^d^	<0.0001	<0.0001
*p* value	0.0002		0.01			
Muscles	9.39 ± 1.84^a^	16.8 ± 1.55^b^	17.8 ± 1.41^b^	21.8 ± 1.92^c^	0.0003	0.006
*p* value	0.0009		0.01			
Testes	11.8 ± 2.89^a^	17.9 ± 1.53^b^	31.0 ± 5.41^c^	26.5 ± 3.09^c^	<0.0001	<0.0001
*p* value	<0.0001		0.04			

**(b):** 

*α*-Tocopherol (nM/g)	Two-way ANOVA, *p* value		
	Training	Vitamin E	Training × vitamin E
Liver	<0.0001	<0.0001	NS
Heart	<0.0001	<0.0001	NS
Muscles	<0.0001	<0.0001	NS
Testes	NS	<0.0001	0.0001

C: control group nonsupplemented with *α*-tocopherol, not trained; CT: control group nonsupplemented with *α*-tocopherol, trained; E: group supplemented with *α*-tocopherol, not trained; ET: group supplemented with *α*-tocopherol, trained; ^1^Tukey HSD post hoc test; ^a-d^the same letters indicate homogenous groups, *p* value ≤0.05; NS: not significant.

**(a):** 

TBARS (nM/g)	Groups				*p* value^1^ C vs. E	*p* value CT vs. ET
	Control		Vitamin E			
	C	CT	E	ET		
	*N* = 8	*N* = 8	*N* = 8	*N* = 8		
Liver	5.08 ± 0.65^a^	4.41 ± 0.41^a^	4.98 ± 0.62^a^	4.70 ± 0.45^a^	NS	NS
*p* value	NS		NS			
Heart	3.90 ± 0.65^a^	4.86 ± 1.03^b^	2.94 ± 0.71^a^	3.52 ± 0.39^a^	NS	0.0032
*p* value	NS		NS			
Muscles	2.84 ± 0.56^a^	5.33 ± 1.26^b^	2.93 ± 0.74^a^	2.61 ± 0.32^a^	NS	<0.0001
*p* value	0.0002		NS			
Testes	1.22 ± 0.31^a^	1.89 ± 0.47^b^	1.49 ± 0.26^a,b^	1.51 ± 0.26^a,b^	NS	NS
*p* value	0.0039		NS			

**(b):** 

TBARS (nM/g)	Two-way ANOVA, *p* value		
	Training	Vitamin E	Training × vitamin E
Liver	0.0158	NS	NS
Heart	0.0047	<0.0001	NS
Muscles	0.0004	<0.0001	<0.0001
Testes	0.0083	NS	0.0145

C: control group nonsupplemented with *α*-tocopherol, not trained; CT: control group nonsupplemented with *α*-tocopherol, trained; E: group supplemented with *α*-tocopherol, not trained; ET: group supplemented with *α*-tocopherol, trained; ^1^Tukey HSD post hoc test; ^a,b^the same letters indicate homogenous groups, *p* value ≤0.05; NS: not significant.

## Data Availability

The data used to support the findings of this study are available from the corresponding author upon request.
